# Utilization of Yeast Extract as a Flavor Enhancer and Masking Agent in Sodium-Reduced Marinated Shrimp

**DOI:** 10.3390/molecules29010182

**Published:** 2023-12-28

**Authors:** Evren Burcu Şen Yılmaz

**Affiliations:** Faculty of Fisheries, Ege University, 35100 Bornova, Izmir, Turkey; evren.burcu.sen@ege.edu.tr; Tel.: +90-5065314684

**Keywords:** sodium replacement, yeast extract, marinated shrimp, NaCl, KCl, amino acid composition, nutritional composition

## Abstract

Deepwater pink shrimp (*Parapenaus longirostris*) has a significantly high catch yield and is a highly important food source for human nutrition in terms of its nutritional value. The reduction of salt content in seafood products while preserving taste poses a significant challenge. The aim of this study is to reduce the NaCl ratio used in the shrimp marination process by substituting it with KCl and masking the resulting bitterness from KCl using natural flavor enhancers, such as yeast extracts. The marinated shrimp were prepared using 50% KCl instead of 50% NaCl. In order to mask the bitter taste caused by KCl and enhance the flavor, two different types of yeast extracts obtained from *Saccharomyces cerevisiae* were utilized in the formulation. Nutritional composition, Na and K contents, amino acid composition, color measurement, bacteriological quality, pH changes, and sensory evaluations were conducted to assess the impact of salt reduction and yeast extracts on the sensory, chemical, and physical attributes of the products. L-glutamic acid, L-alanine, L-aspartic acid, L-leucine, L-valine, and L-lysine were found to be higher in samples with Levex Terra yeast extract. Despite a 50% reduction in NaCl content, the addition of yeast extract led to an increase in the umami taste due to the elevation of amino acids present. Yeast extracts can offer a promising solution for enhancing the sensory qualities of seafood products with reduced salt content by conducting more detailed sensory development examinations.

## 1. Introduction

Salt consumption is a ubiquitous dietary element that has both enriched and imperiled human health on a global scale [1,2]. While salt is an essential component for bodily functions, excessive consumption has emerged as a pervasive public health concern [3]. Excessive salt intake has been linked to a litany of health issues, including hypertension, cardiovascular diseases, and stroke [4,5], making it a formidable global health challenge. This paradoxical nature of salt underscores the critical need for a balanced and informed approach to its consumption, one that acknowledges both its necessity and the potential damage it can inflict on individuals and societies worldwide.

Health organizations around the world have established recommended limits for salt intake to safeguard public health and mitigate the risks associated with excessive sodium consumption. The most widely recognized and endorsed guideline is set by the World Health Organization (WHO), which recommends that adults should consume less than 5 g of salt (approximately 2000 milligrams of sodium) per day [6]. This threshold is primarily aimed at reducing the incidence of hypertension and cardiovascular diseases, which are among the leading causes of mortality globally. Similarly, other prominent health institutions, such as the American Heart Association [7] and the Centers for Disease Control and Prevention in the United States [8], echo these guidelines, emphasizing the importance of limiting daily salt intake to maintain overall well-being. These recommended limits serve as crucial benchmarks, encouraging individuals to be mindful of their dietary choices and take proactive measures to protect their health.

Approximately 70–75% of salt intake in Europe, Australia, and North America is believed to originate from the consumption of processed foods. Therefore, salt reduction in these products is a priority at the international level [6]. The average salt consumption in Turkey was estimated to be higher than the recommended limits set by health organizations, with the daily salt intake for an average Turkish adult being around 18 g, which is significantly above the WHO’s recommended limit of 5 g per day.

In the realm of food science and production, the quest to reduce salt content in various products while preserving taste and consumer acceptance has spurred significant innovation [9]. Innovative approaches in food science propose the utilization of flavor enhancers, such as yeast extracts, to amplify salty taste perception without the need for additional salt. Notably, Zheng et al. [10] conducted a comprehensive study where they successfully isolated, purified, and identified five distinct peptides from yeast extracts, each possessing remarkable salty-enhancing attributes. Sour, sweet, bitter, salty, and umami are the five fundamental tastes that humans perceive, with “salty” being a crucial taste sensation. Presently, the average daily sodium intake per person exceeds twice the recommended levels established by WHO, which advises a daily sodium intake of less than 2 g (or 5 g of salt) [11,12,13,14].

Yeast extract is a complex mixture comprising various components such as proteins, peptides, amino acids, nucleic acids, B vitamins, minerals, carbohydrates, and more [15]. The specific chemical makeup of yeast extracts can vary depending on factors like the conditions under which yeast is cultured and how the yeast extract is prepared. For instance, dried brewer’s yeast, often used as a dietary supplement, is notably high in protein, containing nearly 50 g per 100 g of yeast. It also serves as a source of essential B vitamins (including B1, B2, B3, B5, B6, and B9) and minerals like iron, phosphorus, magnesium, and zinc. Yeast extracts are particularly rich in free amino acids, with glutamic acid, glycine, alanine, and valine being among the most prominent ones [15].

Yeast extracts have garnered significant attention in the food industry for their role as natural flavor enhancers and their potential as masking agents in low-sodium or salt-reduced food products [16]. These extracts, derived from yeast cells through autolysis or enzymatic hydrolysis processes, are rich sources of umami compounds, nucleotides (such as inosinate and guanylate), and free amino acids (including glutamic acid), which collectively contribute to a savory and robust flavor profile often described as umami [15,16]. In the context of masking agents, yeast extracts can be instrumental in mitigating the adverse taste effects associated with salt reduction. When sodium levels are reduced in food formulations, there is a risk of diminished taste perception, and the overall palatability of yeast extracts can help counterbalance this by intensifying the umami and savory notes in foods, effectively masking the absence of salt and enhancing the overall flavor experience [15] This makes them valuable tools for food manufacturers seeking to create healthier products with reduced sodium content while still maintaining the desired taste and consumer appeal. Moreover, yeast extracts have been recognized for their versatility in various culinary applications, including soups, sauces, snacks, and meat products, where they not only serve as flavor enhancers but also as natural alternatives to synthetic flavor enhancers like monosodium glutamate (MSG).

This study attempted to address this aspect of current food technology to examine its effects and contributions to food in the context of salt-reduced food production using masking agents and flavor enhancers. For this purpose, yeast extract was added to reduced-salt marinated shrimp and examined.

## 2. Results

### 2.1. Nutritional Composition, Na and K Content

Moisture, crude fat, crude protein, crude ash, carbohydrate, and energy results of marinated shrimp groups are given in Table 1. No significant difference in moisture content was observed between the control and the experimental groups with the addition of 1% yeast extract. However, the moisture content was found to be significantly (*p* < 0.05) lower in the S3 and T3 with the addition of 3% yeast extract. Although slight differences in crude protein values were detected among the groups, they were not statistically different. The S1, T1, and T3 exhibited significantly (*p* < 0.05) higher crude fat content compared to the C and S3. The energy value of the S3 was detected significantly lower (*p* < 0.05) than other groups, in parallel with the decrease in crude fat content.

During the processing of shrimp marinade, the NaCl levels in both the control and the other four experimental groups were reduced by 50%, and they were replaced with an equal amount of KCl. Results of Na and K minerals are given in Table 2. However, the Na values of the S3, T1, and T3 were found to be significantly higher (*p* < 0.05) compared to the C, whereas only the Na value of the S1 was lower than that of the control group and the other experimental groups (*p* < 0.05). Similarly, the K values of the S3, T1, and T3 were higher than those of the C group, while the K values of the S1 were found to be significantly lower (*p* < 0.05) than those of all other groups.

### 2.2. Amino Acid Composition of Marinated Shrimp

The addition of yeast extract to marinated shrimp has resulted in both an increase and decrease in amino acid values (Table 3). L-alanine, glycine, L-valine, L-leucine, L-isoleucine, L-threonine, L-serine, L-proline, L-phenylalanine, L-histidine, and L-tyrosine amino acids were found to be significantly lower (*p* < 0.05) in the T1 when compared to the control and other experimental groups. In contrast, L-aspartic acid and L-glutamic acid values were significantly higher in this group. In the T3, L-leucine, L-valine, L-alanine, L-isoleucine, L-proline, L-methionine, L-phenylalanine, and L-tyrosine amino acids were found to be significantly higher in T1 samples. Upon examining the amino acid composition data, it is evident that the incorporation of both yeast extract varieties into marinated shrimp has led to a substantial and statistically significant (*p* < 0.05) augmentation in the concentrations of L-arginine and L-lysine amino acids within the marinated shrimp. In the groups where Levex Simplo and Levex Terra were added, as the percentage of added yeast extract increased the amino acid values also showed an increase, with higher levels observed in S3 and T3 containing 3% yeast extract.

### 2.3. Color Measurement of Marinated Shrimp

The addition of both Simplex and Levex yeast extracts at 1% and 3% concentrations has resulted in changes in the color values of marinated shrimp (Table 4). The addition of both types of yeast extracts has led to a slight decrease in the L* values. The highest L* value was found in C (71.04 ± 1.87), while the lowest value was observed in S1 (66.75 ± 3.31). The changes in the a* values are not statistically significant. However, the addition of both types of yeast extracts, particularly, significantly influenced the yellowish tones, as indicated by the b*(+) values, causing an average increase of approximately 10-fold. The b* value for C was 1.68 ± 1.12, whereas the values for groups with yeast extract additions S1, S3, T1, and T3 were found to be 11.60 ± 2.02, 12.88 ± 1.88, 11.19 ± 1.77, and 14.47 ± 1.19, respectively.

### 2.4. Bacteriological Analysis and pH Measurements of Raw and Marinated Shrimp

The initial values of pH and TBC for raw shrimp were 4.56 ± 0.02 log cfu/g, 6.68 ± 0.04, respectively. The TBC values of raw shrimp were found to be below the recommended consumption limit of 7.00 log CFU/g for frozen shrimp (ICMSF, 1992), indicating good quality. While slight variations were observed in the TBC values among the C and other groups with yeast extract additions, none of these were found to be statistically significant (Table 5). When examined in terms of the APC values, it is observed that the values for Groups T1 and T3 are higher than those for C, S1, and S3. However, this difference is not of a magnitude that would pose a bacterial load problem; it represents a rather small increase. The APC values ranged from 2.08 ± 0.13 to 3.00 ± 0.23. In all groups of marinated shrimp, including C, S1, S3, T1, and T3, no statistically significant differences were observed in the ABC values. In all groups of marinated shrimp, pH values were found to be lower than the initial pH value of raw shrimp. All values were below the expected pH of 4.5 for marinades.

### 2.5. Sensory Evaluation of Marinated Shrimp

Sensory evaluation scores for marinated shrimp are presented in Table 6. According to these scores, no significant differences were observed among the groups across all parameters. This lack of distinction can be attributed to some panelists assigning high scores to shrimp marinades with various types and proportions of added yeast extract, while a smaller subset favored the control group. Consequently, the standard deviations were high, preventing clear differentiation between the groups. For example, upon examining the numerical values in the table, it is evident that the T1 group received the highest score in overall acceptability. However, as this difference lacks statistical significance, we cannot deem it a meaningful assessment.

## 3. Discussion

The moisture content of marinated shrimp varied between 53.85 ± 0.70 and 53.06 ± 1.10 g/100 g. Previously, in marinade studies conducted with the same type of shrimp, moisture values of approximately 75–80% were reported [17,18,19]. The reason for obtaining a different result in this study is attributed to the analysis being conducted after marinated shrimp were packaged in sunflower oil. Another contributing factor to this outcome is the crude fat content. In this study, the crude fat content was found to be between 27% and 32%, whereas in other shrimp studies, fat content was reported to be approximately 0.5% to 5% [17,18,19]. In another study conducted with the species *Parapenaus longirostris*, the values for crude protein, moisture, crude fat, and crude ash were found to be 20.70 ± 0.03, 75.30 ± 0.08, 1.0 ± 0.03, and 2.2 ± 0.05, respectively [18]. In the current study, the protein content was determined to be around 13 g/100 g, whereas, in other shrimp marinaded studies, it was reported as 16 g/100 g [19].

The sodium content of marinated shrimp, in which potassium salt was used instead of sodium salt, resulting in a 50% reduction in sodium, was found to be 2189.97 mg/kg in the C, while the lowest sodium content was observed in S1 at 2034.33 mg/kg. The yeast extract company Levex has reported that the salt content in yeast extracts is process-dependent, with percentages being 0–6% for Simplo and 28% for Terra. It is believed that the type and percentage of yeast extracts used for masking the bitter taste derived from potassium and enhancing flavor have influenced both sodium (Na) and potassium (K) values. In a study where authors examined certain properties of different yeast extracts they produced, the authors reported that the Na content ranged from 791.84 ± 11.63 to 2288.55 ± 42.3 mg/100 g [20]. In the same study, it was reported that K levels varied between 3532.71 ± 57.53 and 4558.51 ± 66.42 mg/100 g. In a study where authors examined certain properties of different yeast extracts they produced, the authors reported that the sodium (Na) content ranged from 791.84 ± 11.63 to 2288.55 ± 42.3 mg/100 g [20]. The same study reported that potassium (K) levels varied between 3532.71 ± 57.53 and 4558.51 ± 66.42 mg/100 g. Dried yeast and yeast extract paste were examined, and the calcium content was determined to be 130.86 mg/100 g, and potassium levels were reported as 2460.2600 mg/100 g, respectively [15].

The main component of yeast extracts is partly hydrolyzed protein [21]. Yeast extracts usually contain a high degree of free amino acids and group B vitamins. Free amino acids include primarily glutamic acid, glycine, alanine, and valine [15]. In another study conducted by obtaining three different yeast extracts from each of two different yeast types, the amino acid composition of yeast extracts was determined [20]. According to this, the amino acids with the highest average values among the six types of yeast extracts, ranked from highest to lowest, are glutamic acid, alanine, aspartic acid, leucine, valine, lysine, and threonine [20]. In both of the aforementioned studies, the amino acids that are known to be high in yeast extracts were found to be higher in the groups primarily containing Levex Terra yeast extract added groups when compared with Lexex Simplo groups in the present study. Especially in the groups where 3% yeast extract was added, a more significant (*p* < 0.05) increase in amino acids was observed compared to the groups where 1% yeast extract was added.

The addition of yeast extract has caused changes in the color L*, a*, and b* values of marinated shrimp. The brightness values of shrimp meat were significantly decreased, particularly in the S, where yeast extract was added. Campagnol et al. [22] and Corral et al. [23] examined different properties of sausages produced from pork meat with low salt content and the addition of yeast extract. In both studies, they reported that yeast extract increased the L*, a*, and b* values of the sausages. The decrease in the L* value in this study, while L* values increased slightly in the previous two studies, is believed to be due to differences in the color of the raw materials used in the products. In the two previous studies, the raw material used for sausages was pork meat, whereas for marinated shrimp, the color of the shrimp species used was closer to pale, whitish pink. Therefore, yeast extracts must have imparted different color shades to products with different raw materials. Furthermore, while yeast extracts in pork sausages increased the b* values to a low extent in both studies, in marinated shrimp samples, they led to an increase of approximately tenfold (*p* < 0.05). In a marinade study conducted with *Parapenaus longirostris*, marinades were prepared using NaCl, no additives were used for the control group, and they were stored in a refrigerator for 75 days [18]. During storage, L* values were reported to vary between 49 and 73. In the same study, the a* values of marinated shrimp from the control group were reported to range between 0.8 and 1.9, while the b* values ranged from 6.5 to 10. Marinated shrimp were prepared using *Parapenaus longirostris*, and six different marination temperatures were utilized, followed by color analyses conducted after storage in a brine solution [24]. According to the color measurement results of that study, L* values were determined to be in the range of 76.17 to 79.72, a* values ranged from 1.40 to 4.97, and b* values ranged from 6.80 to 9.41. The color of shrimp is quite different for different species and is influenced by different parameters such as feed, season, and environment. The carotenoid content in wild shrimp can vary based on their natural habitat and the presence of algae, which are the primary producers of carotenoids in aquatic environments [25]. Hence, it is anticipated that the color parameters of shrimp meat will vary even in similar studies conducted with the same species at different times and locations.

The initial bacteriological quality of frozen shrimp was detected by the TBC, which was 4.56 ± 0.02 log CFU/g. The TBC did not exceed the maximum limit (7 log CFU/g) of bacteriological criteria for frozen shrimp given by ICMSF (1992). The initial bacteriological quality of the frozen shrimp was given 5.24 log CFU/g for total aerobic bacteria count [17]. According to another study on shrimp croquettes, the initial value was found to be 3.49 log CFU/g [18]. According to another study on frozen shrimp, the initial aerobic bacterial count reported was 4 log CFU/g [26], and all the results are consistent with the findings of this study. No significant difference was found in the TBC results among the shrimp marinade groups with reduced salt and the addition of yeast extract (C, S1, S3, T1, T3). The TBC values for the groups were found to range between 2.96 and 3.22 log CFU/g. These values are below the consumption limit specified by ICMSF [27] (6 log CFU/g). In different shrimp marinade studies, the TBC values obtained from control groups were reported as 1.93 and 1.75 log CFU/g, respectively, by Cadun et al. [17] and Cadun et al. [18]. In this study, the PC values were determined as 2.08, 2.53, 2.11, 3.00, and 2.61 for C, S1, S3, T1, and T3, respectively, and there was no statistically significant difference between these values. When examining the AC data of the marinated shrimp, no significant difference was observed among the groups, with values ranging from 2.11 to 2.35 log CFU/g.

The pH changes occurring in seafood after fishing can be used as a quality criterion [28]. In a study investigating the relationship between the pH values of shrimp and their quality, a quality index was created based on pH values, where pH < 7.7 was reported as “Good”, 7.7–7.95 as “Consumable”, and >7.95 as “Not Consumable” [29]. According to that index, the pH ratio of the frozen raw shrimp in this study was found to be 6.68 and of good quality. Similarly, in another study conducted with the *Parapenaus longirostris*, the initial pH was reported as 7.01 [30]. The pH value should not be more than 4.8 in marination [31]. After the shrimp marinated were prepared in the study, the pH values for C, S1, S3, T1, and T3 were determined as 4.30, 4.28, 4.06, and 3.99, respectively. The types of yeast extract and the amounts added did not statistically affect the pH value. The pH values in all groups are below the pH limit values specified for marinades. Similar results were obtained for the same shrimp species; the pH values for control groups were reported as 4.2 and 4.61, respectively [17,18].

NaCl is extensively employed in seafood preparation and processing, serving as both a preservative agent by inhibiting microbial growth and a flavor modifier due to its contribution to a salty taste. Furthermore, it enhances the sensory characteristics of other components, including texture and color, while also serving as a binding and emulsifying agent [32]. While NaCl plays such a crucial role in the sensory development of seafood products, replacing a portion of NaCl with an alternative salt like KCl, although making the product healthier, can lead to sensory drawbacks, such as the bitter taste derived from potassium. Regarding the acceptability test parameters (e.g., the overall color, texture, and flavor), 25–50% of KCl was at least applicable as a control [33,34]. The increase in such reports has led to the growing use of natural flavor enhancers like the yeast extracts employed in this study, particularly to mitigate potential losses in sensory parameters, especially taste, in recent years. Various studies have indicated that substituting NaCl with KCl may lead to the development of bitter or metallic off-flavors [35,36,37,38]. Results of a previous study showed that the replacement of NaCl by KCl up to a 50:50 ratio does not significantly change the sensory quality (color, taste, odor, texture, and overall quality) of salt replaced marinated anchovy but taste presented an unpleasant bitter taste [39]. Furthermore, Fuentes et al. [39] reported that the taste scores significantly decreased in smoked sea bass samples when the KCl content exceeded 50%. In the current study, marinated shrimp with a 50% reduction in NaCl content have utilized KCl. Yeast extracts have been employed to reduce the perception of the bitterness arising from KCl and to enrich the taste. The preparation methods of yeast extracts, as well as the interactions between amino acids, nucleotides, carbohydrates, and peptides present in the extracts, can lead to a wide range of flavors, along with the production of volatile compounds [15]. In the present study, when the results of sensory evaluation were assessed, the numerical values of the groups differed in color, flavor, less bitter taste, texture, and overall acceptability parameters; however, this did not lead to a statistically significant difference. Among the parameters of color, taste, less bitter taste, texture, and overall acceptability, the highest values were obtained by the T1, which contained 1% Levex Terra extract; however, this is not a statistically significant difference. The reduction of salt by 50% in sausages has led to a decrease in the sensory scores of the samples. However, the addition of 2% yeast extract has been shown to improve sensory quality and increase ratings, mitigating the adverse effects of KCl [22]. Corral et al. [23] reported that inoculating yeast extract into wild boar sausages resulted in the absence of wild boar odor perception and contributed to sensory enhancement. Based on the sensory evaluation results of marinated shrimp, it can be concluded that using a small amount of yeast extract was sufficient to enhance the appeal and flavor of shrimp marinade products with 50% reduced salt content.

## 4. Materials and Methods

### 4.1. Materials

Frozen shrimp (*Parapenaus longirostris*) were obtained from a seafood processing facility in İzmir/Türkiye. Boxes were transferred to the laboratory under a cold chain and kept at −20 °C until used for marination. After the marinating process, shrimp was placed in separate plastic containers with sunflower oil obtained from a market in İzmir/Turkey. Yeast extracts (Levex Simplo and Levex Terra) were supplied by Levex Company (İstanbul, Türkiye). The company obtains yeast extracts from the *Saccharomyces cerevisiae* species. The nutritional composition, Na content, and pH values of both types of yeast extracts are shown in Table 7.

### 4.2. Marination Process

Frozen shrimp were put into heat-resistant plastic bags and boiled for 10 min, then cooled immediately in iced water. The shrimp were then separated into groups and put into the brine. All groups’ brine included 2% acetic acid, 2% sodium chloride, 2% potassium chloride, and yeast extract (except the control group). The shrimp:brine ratio was 1:1 (*w*/*v*). In the experimental group’s brine, 1% and 3% of Levex Simplo or Levex Terra yeast extracts were used individually. The brine contents, according to the groups, are provided below: 

Control group (C): 2% acetic acid, 4% chloride (50% NaCl + 50% KCl);

Including Levex Simplo as a masking agent groups:

(S1): 2% acetic acid + 4% chloride (50% NaCl + 50% KCl) + 1% Levex Simplo;

(S3): 2% acetic acid + 4% chloride (50% NaCl + 50% KCl) + 3% Levex Simplo.

Including Levex Terra as a masking agent groups:

(T1): 2% acetic acid + 4% chloride (50% NaCl + 50% KCl) + 1% Levex Terra;

(T3): 2% acetic acid + 4% chloride (50% NaCl + 50% KCl) + 3% Levex Terra.

Shrimp was kept for 48 h at 4 °C in brine, then drained and put into a plastic container with equal sunflower oil. The samples were kept at 4 °C during the analyses and were conducted immediately.

### 4.3. Nutritional Composition, Sodium (Na), and Potassium (K) Content

Moisture [40], crude fat [41], crude protein [42], crude ash [43], carbohydrate, and energy [44] analyses were performed to determine the nutrition composition of the shrimp. Sodium and potassium contents were determined with the ICP-MS using the AOAC method [45]. All analyses were undertaken at the TUBITAK Marmara Research Center, Food Institute, Gebze, Turkey.

### 4.4. Amino Acid Composition

Amino acid analysis was performed using the hydrolyze method. Eppendorf LC 3000 was performed using an amino acid analyzer manual according to the IUPAC gas chromatograph method [46]. All analyses were undertaken at the TUBITAK Marmara Research Center, Food Institute, Gebze, Turkey.

### 4.5. Color Measurement

Color measurements were carried out using a Spectro Pen^®^ (Hach-Lange GmbH & Co., Dusseldorf, Germany), making color measurements using the CIE system. In the CIE L*a*b* system, L* denotes lightness on a scale from 0 to 100 from black to white; a* denotes (+) red or (−) green, and b* denotes (+) yellow or (−) blue [47].

### 4.6. Bacteriological Analysis

Raw shrimp samples and marinated shrimp were analyzed for total aerobic bacterial counts (TBC log CFU/g). Also, marinated shrimp were analyzed for psychrotrophic bacterial counts (PBC MPN/mL) and anaerobic bacteria counts (ABC log CFU/g). A portion of 25 g of the sample was weighed and mixed with 225 mL of peptone water (1:10 dilution). A sample mixture was homogenized by using a stomacher (IUL Instruments, Barcelona, Spain) for 1 min at 230 rpm. The TBC was counted on plate count agar, followed by incubation for 24 to 48 h at 30 °C [48]. The PBC was counted on plate count agar, followed by incubation for 7 to 10 days at 7 °C [49]. For the ABC, plate count agar was used, and plates were incubated at 35 °C for 48 h in the anaerobic jar [27].

### 4.7. Sensory Analysis

Sensory analysis was performed to determine the effect of yeast extracts (Simplo and Terra) as masking agents on the bitter taste that resulted from using KCl in the salt reduction process as a replacement for NaCl. Sensory evaluation was carried out by 10 well-trained panelists, according to Gisela [50], with slight modification. A less bitter test parameter was added as a slight modification to identify taste from KCl.

### 4.8. Statistical Analysis

Statistical analysis was performed using IBM SPSS (statistical package for the social sciences) Statistics 22.0 (IBM, New York, NY, USA) and expressed as mean ± SD of the three replicated analyses. One-way analysis of variance (ANOVA) was applied to the data, where parametric assumptions for multiple comparisons were met. In groups where a difference was found with this test, the post hoc Tukey HSD test and Duncan test were employed to identify the source of the difference. Assessments of intergroup parameters and relationships between parameters were considered significant at *p* < 0.05. All analyses except initial total bacteria count and pH were carried out after the shrimp was marinated and packaged in sunflower oil. All analyses were undertaken three times.

## 5. Conclusions

In conclusion, this study highlights the potential of yeast extracts to mitigate sensory losses associated with reduced salt content in marinated shrimp while maintaining bacteriological safety. These findings contribute to the development of healthier and more appealing seafood products, addressing the global concern of excessive salt intake. In the sodium-reduced product, Levex Terra yeast extract, which naturally has higher sodium content than Levex Simplo, is preferred to be added in low percentages. It also emphasizes the necessity for more detailed and comprehensive sensory evaluations in future studies on yeast extract, involving broader participation.

## Figures and Tables

**Table 1:** Nutritional composition results of marinated shrimp.

Analyses	C	S1	S3	T1	T3
Moisture (%)	55.90 ± 0.9 ^ab^*	56.40 ± 0.40 ^b^	53.85 ± 0.70 ^ac^	55.49 ± 1.0 ^ab^	53.06 ± 1.1 ^c^
Crude protein (%)	12.63 ± 0.9 ^a^	12.13 ± 0.80 ^a^	13.72 ± 0.31 ^a^	12.06 ± 1.2 ^a^	13.13 ± 0.8 ^a^
Crude fat (%)	29.87 ± 1.1 ^ac^	32.37 ± 0.67 ^b^	27.49 ± 0.86 ^c^	30.62 ± 0.8 ^ab^	31.20 ± 0.5 ^b^
Carbohydrate (%)	0.06 ± 0.08 ^a^	0.06 ± 0.05 ^a^	0.49 ± 0.05 ^b^	0.09 ± 0.001 ^a^	0.72 ± 0.05 ^c^
Crude ash (%)	1.54 ± 0.8 ^a^	1.69 ± 0.36 ^a^	1.82 ± 0.29 ^a^	1.74 ± 0.4 ^a^	1.89 ± 0.2 ^a^
Energy kcal/100 g	320 ± 1.8 ^a^	339.00 ± 2.60 ^b^	305.0 ± 01.89 ^c^	324 ± 1.0 ^a^	336 ± 0.9 ^b^

*: Means in the same row with the same letter do not differ significantly at the *p* > 0.05 significance level. C: control; S1: 1% Levex Simplo yeast extract added; S3: 3% Levex Simplo yeast extract added; T1: 1% Levex Terra yeast extract added; T3: 3% Levex Terra yeast extract added.

**Table 2:** Na and K contents of marinated shrimp.

Minerals	C	S1	S3	T1	T3
Na mg/kg	2189.97 ± 2.85 ^a^*	2034.33 ± 2.08 ^b^	2636.97 ± 6.35 ^c^	2514.00 ± 2.00 ^d^	3010.07 ± 1.80 ^e^
K mg/kg	2952.00 ± 4.00 ^a^	2896.67 ± 3.51 ^b^	3365.33 ± 5.51 ^c^	3127.60 ± 4.91 ^d^	3064.30 ± 1.67 ^e^

*: Means in the same row with the same letter do not differ significantly at the *p* > 0.05 significance level. C: control; S1: 1% Levex Simplo yeast extract added; S3: 3% Levex Simplo yeast extract added; T1: 1% Levex Terra yeast extract added; T3: 3% Levex Terra yeast extract added.

**Table 3:** Amino acid compositions of marinated shrimp.

Amino Acids mg/100 g	C	S1	S3	T1	T3
L-alanine	723.13 ± 1.11 ^a^*	712.17 ± 7.94 ^b^	773.23 ± 1.59 ^c^	555.73 ± 1.42 ^d^	824.77 ± 2.66 ^e^
Glycine	578.10 ± 1.95 ^a^	467.57 ± 2.28 ^b^	484.93 ± 2.60 ^c^	350.53 ± 3.13 ^d^	573.33 ± 1.53 ^a^
L-valine	629.87 ± 2.20 ^a^	595.63 ± 1.19 ^b^	616.00 ± 2.60 ^c^	451.90 ± 3.25 ^d^	694.07 ± 1.20 ^e^
L-leucine	1060.03 ± 1.95 ^a^	995.20 ± 3.00 ^b^	1060.07 ± 2.90 ^a^	767.37 ± 2.47 ^c^	1195.87 ± 3.10 ^d^
L-isoleucine	712.00 ± 2.20 ^a^	663.90 ± 2.85 ^b^	680.90 ± 1.75 ^c^	485.97 ± 2.95 ^d^	763.87 ± 3.87 ^e^
L-theronine	504.37 ± 5.46 ^a^	586.00 ± 2.00 ^b^	777.30 ± 2.86 ^c^	476.70 ± 3.06 ^d^	491.10 ± 1.95 ^e^
L-serine	550.77 ± 2.36 ^a^	719.93 ± 2.30 ^b^	923.27 ± 1.91 ^c^	504.21 ± 5.76 ^d^	600.63 ± 2.74 ^e^
L-proline	318.37 ± 1.48 ^a^	320.13 ± 1.90 ^b^	338.83 ± 4.02 ^b^	259.67 ± 4.41 ^c^	360.07 ± 1.40 ^d^
L-arginine	595.10 ± 2.85 ^a^	729.23 ± 1.17 ^b^	908.60 ± 8.62 ^c^	619.90 ± 2.25 ^d^	667.10 ± 1.85 ^e^
L-aspartic acid	1369.80 ± 6.9 ^a^	637.67 ± 2.32 ^b^	769.43 ± 6.46 ^c^	1819.07 ± 1.90 ^d^	1092.00 ± 2.00 ^e^
L-methionine	345.07 ± 0.90 ^a^	376.37 ± 1.48 ^b^	259.50 ± 4.67 ^c^	272.20 ± 1.71 ^d^	353.07 ± 1.80 ^e^
L-glutamic acid	2189.77 ± 4.35 ^a^	1324.87 ± 2.20 ^b^	2060.33 ± 2.52 ^c^	2144.03 ± 2.05 ^d^	3733.33 ± 1.53 ^e^
L-phenylalanine	664.23 ± 1.27 ^a^	640.13 ± 2.10 ^b^	669.97 ± 0.95 ^c^	468.37 ± 2.07 ^d^	730.03 ± 1.95 ^e^
L-lysine	979.77 ± 2.36 ^a^	1437.73 ± 4.41 ^b^	1421.17 ± 1.76 ^b^	1265.17 ± 1.76 ^c^	1574.60 ± 3.34 ^d^
L-histidine	425.40 ± 2.03 ^a^	409.00 ± 3.30 ^b^	455.10 ± 2.25 ^c^	225.23 ± 0.78 ^d^	338.20 ± 1.11 ^e^
L-tyrosine	520.30 ± 1.87 ^a^	534.17 ± 2.15 ^b^	537.83 ± 1.26 ^b^	396.20 ± 1.21 ^c^	579.90 ± 1.75 ^d^

*: Means in the same row with the same letter do not differ significantly at the *p* > 0.05 significance level. C: control; S1: 1% Levex Simplo yeast extract added; S3: 3% Levex Simplo yeast extract added; T1: 1% Levex Terra yeast extract added; T3: 3% Levex Terra yeast extract added.

**Table 4:** Color measurement results of marinated shrimp.

Analyses	C	S1	S3	T1	T3
L*	71.04 ± 1.87 ^a^*	66.75 ± 3.31 ^b^	66.85 ± 2.85 ^b^	69.12 ± 1.26 ^ab^	68.89 ± 2.39 ^ab^
a*	7.05 ± 1.49 ^a^	7.03 ± 1.17 ^a^	5.49 ± 1.26 ^a^	5.55 ± 0.81 ^a^	6.46 ± 1.0 ^a^
b*	1.68 ± 1.12 ^a^	11.60 ± 2.02 ^ab^	12.88 ± 1.88 ^bc^	11.19 ± 1.77 ^ab^	14.47 ± 1.19 ^c^

*: Means in the same row with the same letter do not differ significantly at the *p* > 0.05 significance level. C: control; S1: 1% Levex Simplo yeast extract added; S3: 3% Levex Simplo yeast extract added; T1: 1% Levex Terra yeast extract added; T3: 3% Levex Terra yeast extract added.

**Table 5:** Bacteriological results and pH values of marinated shrimp.

Analyses	C	S1	S3	T1	T3
TBC log CFU/g	2.96 ± 0.40 ^a^*	3.03 ± 0.31 ^a^	3.22 ± 0.08 ^a^	3.13 ± 0.14 ^a^	2.81 ± 0.19 ^a^
PC log CFU/g	2.08 ± 0.13 ^a^	2.53 ± 0.12 ^a^	2.11 ± 0.14 ^a^	3.00 ± 0.23 ^b^	2.61 ± 0.15 ^b^
ABC log CFU/g	2.35 ± 0.20 ^a^	2.18 ± 0.15 ^a^	2.22 ± 0.17 ^a^	2.25 ± 0.21 ^a^	2.11 ± 0.09 ^a^
pH	4.30 ± 0.11 ^a^	4.28 ± 0.06 ^a^	4.06 ± 0.06 ^a^	4.26 ± 0.13 ^a^	3.99 ± 0.19 ^a^

*: Means in the same row with the same letter do not differ significantly at the *p* > 0.05 significance level. C: control; S1: 1% Levex Simplo yeast extract added; S3: 3% Levex Simplo yeast extract added; T1: 1% Levex Terra yeast extract added; T3: 3% Levex Terra yeast extract added.

**Table 6:** Sensory evaluation of marinated shrimp.

Analyses	C	S1	S3	T1	T3
Color	3.00 ± 0.89 ^a^*	4.17 ± 0.75 ^a^	3.33 ± 1.03 ^a^	4.22 ± 0.72 ^a^	3.85 ± 1.01 ^a^
Taste	3.00 ± 0.6 ^a^	3.50 ± 1.0 ^a^	3.00 ± 1.1 ^a^	3.81 ± 0.92 ^a^	2.96 ± 1.00 ^a^
Odor	4.17 ± 0.75 ^a^	3.17 ± 0.75 ^a^	2.17 ± 0.98 ^a^	3.21 ± 0.85 ^a^	2.43 ± 0.85 ^a^
Less bitter taste	2.00 ± 0.63 ^a^	3.17 ± 0.75 ^a^	3.00 ± 1.10 ^a^	3.10 ± 0.73 ^a^	3.02 ± 0.98 ^a^
Texture	4.33 ± 0.82 ^a^	4.67 ± 0.52 ^a^	4.50 ± 0.84 ^a^	4.70 ± 0.78 ^a^	4.48 ± 1.02 ^a^
Overall acceptability	2.17 ± 0.98 ^a^	4.17 ± 0.98 ^a^	3.33 ± 1.21 ^a^	4.28 ± 1.10 ^a^	3.28 ± 1.12 ^a^

*: Means in the same row with the same letter do not differ significantly at the *p* > 0.05 significance level. C: control; S1: 1% Levex Simplo yeast extract added; S3: 3% Levex Simplo yeast extract added; T1: 1% Levex Terra yeast extract added; T3: 3% Levex Terra yeast extract added.

**Table 7:** Nutritional composition, pH, and Na contents results of yeast extracts.

Analyses	Levex Simplo	Levex Terra
Moisture (%)	2.89	3.73
Crude protein (%)	6.14	38.79
Crude fat (%)	0.16	1.93
Carbohydrate (%)	21.5	16.1
Crude ash (%)	14.1	37.13
Energy kcal/100 g	333	242
Dietary fiber	0.00	2.28
pH	6.49	6.12
Na mg/kg	4108	115,023

Source: Levex Company, unpublished.

## Data Availability

Data are contained within the article.
